# Lung Ultrasound in Patients With Dyspnea From Infective Lung Disease

**DOI:** 10.3389/fmed.2021.709239

**Published:** 2021-08-05

**Authors:** Pierluigi Bracciale, Salvatore Bellanova, Cristiana Cipriani

**Affiliations:** ^1^Pneumology and Respiratory Semi-intensive Care Unit, Covid Center Ostuni Hospital, Brindisi, Italy; ^2^Department of Clinical, Internal, Anesthesiological, and Cardiovascular Sciences, Sapienza University of Rome, Rome, Italy

**Keywords:** ultrasound, lung, dyspnea, pneumonia, emergency

## Abstract

Infective lung disease is a spectrum of pulmonary disorders with high prevalence in clinical practice. In the last decade, many studies focused on the clinical usefulness of lung ultrasound (LUS) in the management of patients presenting with dyspnea from infective lung disease. We report data on the methodological and standardized use of bedside LUS in the differential diagnosis of patients with acute dyspnea from infective lung diseases. We performed a cross-sectional study in 439 patients (160 women and 279 men, mean age 64.2 ± 11.5 years, age range 23–91 years) with infective lung diseases. A bedside LUS with a convex probe and chest X-ray were performed in all subjects. Chest CT was performed in a subgroup of patients, as clinically needed. We observed a statistically significant difference in the percentage of pleural effusion and pulmonary consolidation assessed by LUS, compared to X-ray (52.7 vs. 20%, respectively, *p* < 0.05; 93.6 vs. 48.2%, *p* < 0.001). The majority of the consolidations detected by LUS were mixed, hypo- and hyperechoic, lesions, with air bronchogram in 40% of cases. All findings assessed by LUS were confirmed by chest CT, when performed. We describe the actual role of LUS in the assessment of patients with infective lung disease. It has higher sensitivity compared to chest X-ray in the detection of pleural effusion. Consolidations from infective lung disease have mostly mixed echogenicity by LUS.

## Introduction

Infective lung disease is a spectrum of pulmonary disorders with high prevalence in clinical practice and a very common cause of hospital admission for dyspnea. The most recent evidence, reported in the pre-COVID 19 era, showed an annual hospitalization rate for community-acquired pneumonia (CAP) among US adults ≥65 years ranging from 847 to 3500 per 100,000 persons (1). Clinical practice guidelines recommend that clinical assessment, laboratory findings, and chest imaging should be considered in the diagnosis of CAP in adults (2).

Among imaging technologies, chest radiography (X-ray), lung ultrasound (LUS), and chest high-resolution computed tomography (HRCT) are routinely employed in clinical practice.

Lung ultrasound demonstrated in the last decade to be of significant value in the clinical assessment of different pleuro-pulmonary disorders (3, 4). It is a complementary diagnostic tool, and a valuable guide for both diagnostic and therapeutic interventional procedures (5, 6). In the last decade, many studies focused on the role of LUS in the early differential diagnosis of patients with acute dyspnea from different causes (7–9). In particular, LUS demonstrated to have a valuable accuracy, in combination with clinical findings, in the diagnosis of pneumonia (10, 11). Several studies demonstrated this potential of LUS in both CAP and ventilator-associated pneumonia; emerging evidence suggest a role in the COVID-19 pneumonia, as well (10). Previous studies in a large number of patients demonstrated that the best clinical application of LUS in patients with CAP is in association with X-ray, clinical and laboratory findings, and in patient's monitoring (11, 12). Additionally, the application of the contrast-enhanced ultrasound (CEUS) technique to the study of lungs has no role in the differential diagnosis of CAP vs. lung cancer (13).

One of the most important and recognized application of LUS in clinical practice is for the detection and characterization of pleural effusions and as a guide for thoracentesis (14). The routine use of LUS in the assessment of pleural effusion before and during the execution of the thoracentesis demonstrated to significantly reduce the rate of thoracentesis-related complications (14, 15). Historical data reported a 0.5% occurrence of pneumothorax in case of LUS-guided thoracentesis, compared to a prevalence ranging from 7 to 15% when LUS is not used (16). Finally, LUS is a valuable technique as guidance of percutaneous transthoracic needle biopsy, with a rate of complications <0.5% in our case series (6, 17).

To our knowledge, there have been few reports on the systematic, methodological, and standardized use of bedside LUS, in association with clinical and laboratory findings, and chest X-ray, in the clinical assessment of patients presenting with acute dyspnea from infective lung disease. We report data on this issue gained by a multicenter study involving a large number of patients admitted in different medicine units. Description of LUS semeiotics and its usefulness in clinical practice are provided.

## Materials and Methods

We performed a multicenter cross-sectional study in patients admitted for infective lung disease.

Written informed consent by patients and/or their proxies plus the attending physicians' authorization to perform LUS and chest X-ray was obtained in any patient. The protocol was performed in accordance with the ethical standards laid down in the 1964 Declaration of Helsinki and its later amendments and was approved by the Ethics Committees of all the centers participating the study.

Inclusion criteria were: acute dyspnea from infective lung disease, whose diagnosis was posed in accordance to the Infectious Diseases Society of America/American Thoracic Society guidelines (18). Patients were recruited from the emergency department, intensive care, rehabilitation, internal medicine, and geriatric units. Exclusion criteria were: severe hemorrhage, metabolic acidosis, massive pulmonary embolism, any type of brain injury and any psychogenic cause of dyspnea. Patients' recruitment was performed from September 2017 to September 2019.

At admission, all patients underwent bedside posterior-anterior chest X-ray, and LUS examination. The bedside LUS was performed by internal medicine and geriatric specialists, ICU physicians, pneumologists, or radiologists. All of them had a minimum of 5 (mean 7) years of experience in this technique. Each examination was done with a low–medium frequency (3.5–5 MHz) convex probe. The ultrasound scanners used were: Technos MPX, My Lab30 Gold (Esaote, Genoa, Italy); Aplio XG and Xario XG (Toshiba, Tokyo, Japan). They were all used with the following settings for transthoracic study: depth of images (penetration): 7–14 cm; gain control: 50–60%; use of harmonic imaging; electronic focus: pleural line. The devices used had no more than 7 years of use and annual maintenance was done. Participants were examined in the sitting position and scans were made bilaterally through the ventral (along the parasternal, mid-clavicle and anterior axillary lines), lateral (along the mid-axillary line), and posterior (along the posterior axillary and paravertebral lines) intercostal spaces. Each hemithorax was examined from the apex to the base. Operative time for any LUS exam was about 10 min.

The LUS parameters considered for each lung were: pleural effusion, pleural sliding or gliding sign, pleural line thickening, subpleural nodule, and/or consolidation. For each lung, the examiner recorded the presence and the number of B-lines, as previously described (9).

Video recordings of any exam were reviewed by a second group of operators with a 20-year experience in LUS. None of them had been involved in the original exams, and all were blinded to the outcomes of those studies. The reviewers recorded the presence of the abovementioned LUS parameters and their findings were compared with those of the original operators to assess inter-operator variability.

LUS and X-ray findings were compared with the final diagnosis as made by the physicians in charge of any patient on the basis of clinical guidelines (18). Chest HRCT was performed in a subgroup of patients in order to make the final diagnosis, as needed.

### Statistical Analysis

The percentage of different causes of dyspnea assessed by LUS and X-ray were compared to the definitive diagnosis using McNemar's test. Findings obtained by original examiners and reviewers were analyzed separately. Inter-operator concordance, in terms of number of different causes of dyspnea, was assessed using a generalized linear mixed-effects model accounting for the double-nested design of data, and inter-examiner variability was tested with the Roy approach (16). *P*-values <0.05 were considered significant. All analyses were performed using SAS® software, release 9.1 (2011 SAS Institute Inc, Cary, NC, USA).

## Results

We studied 439 patients (160 women and 279 men, mean age 64.2 ± 11.5 years, age range 23–91 years). Table 1 reports clinical characteristics of patients, LUS, and chest X-ray findings.

**Table 1:** Clinical characteristics of patients with dyspnea, LUS and chest X-ray findings (*n* = 439).

**Age**, years (mean ± SD)	64.2 ± 11.5
**Female/male ratio**	1/1.7
**Current smoking**, number of patients (% of the total)	319 (72.6)
**Comorbidities**, number of patients (% of the total)	239 (54.5)
**LUS**	
Pleural effusion, number of patients (% of the total)	231 (52.7)
Lung consolidation, number of patients (% of the total)	411 (93.6)
Hyperechoic spots (“air bronchogram”), number of patients (% of the total)	176 (40)
Pleural effusion and lung consolidation, number of patients (% of the total)	224 (51)
B-lines >3, number of patients (% of the total)	439 (100)
**Chest X-ray**	
Pleural effusion, number of patients (% of the total)	88 (20)
Lung consolidation, number of patients (% of the total)	211 (48.2)
Air bronchogram, number of patients (% of the total)	41 (9.3)
Pleural effusion and lung consolidation, number of patients (% of the total)	32 (7.2)

**Figure 1:**
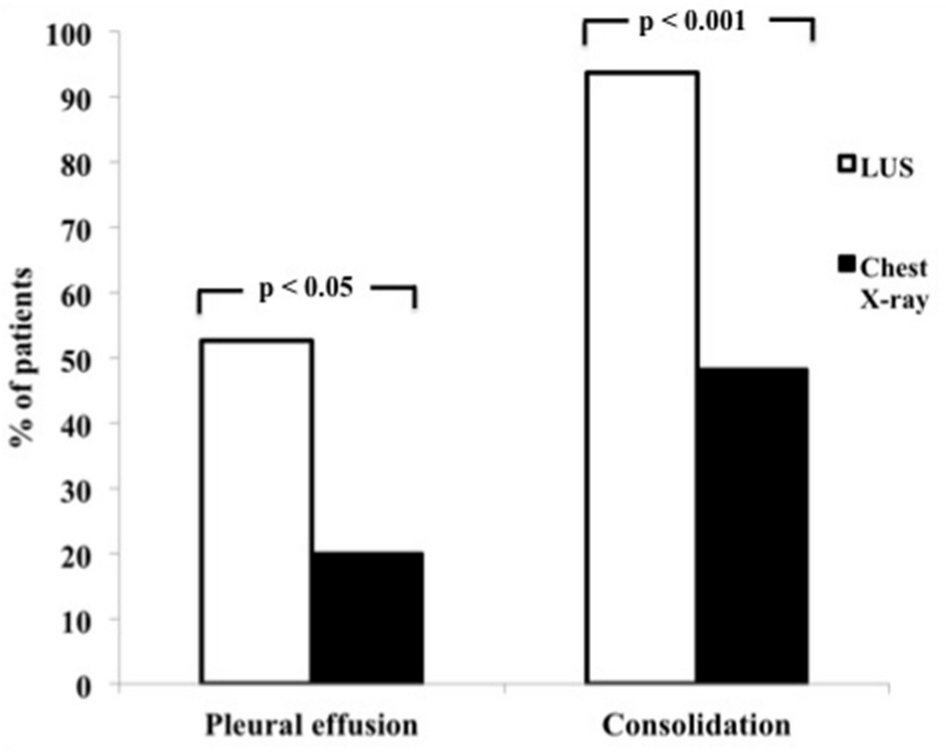
Percentage of pleural effusion and lung consolidation assessed by LUS and chest X-ray.

As shown, the proportion of patients with pleural effusion alone or pleural effusion in association with lung consolidation detected by LUS was higher than by chest X-ray. In particular, there was a statistically significant difference between the two techniques in the percentage of pleural effusion (52.7 vs. 20%, *p* < 0.05 by McNemar's test) (Figure 1).

Pulmonary consolidation was found in 93.6% of patients by LUS and in 48.2% by chest X-ray (*p* < 0.001 by McNemar's test) (Figure 1).

A number of B-lines >3 was detected by LUS in all patients (Table 1).

Table 2 reports ultrasound semeiotics of the lesions assessed by LUS. A single consolidation was found in 417 patients (95% of cases) and multiple consolidations in the remaining 22 (5%) of cases. The majority of the consolidations detected by LUS was localized in the posterior-basal and lateral-basal regions and were 2–3 cm of main diameter (Table 2). They were mostly mixed, hypo- and hyperechoic, lesions, with hyperehoic spots (“air bronchogram”) visualized by LUS in 40% of cases (Tables 1, 2).

**Table 2:** Ultrasound semeiotics of lung consolidation.

Single, number of patients (% of the total)	417 (95)
Multiple, number of patients (% of the total)	22 (5)
**Localization, number of patients (% of the total)**	
Anterior-apical	15 (3.4)
Posterior-basal	166 (38)
Lateral-basal	121 (27.6)
Lateral-medial	63 (14.2)
Posterior-medial	55 (12.5)
Anterior-medial-lateral	19 (4.3)
**Main diameter, number of patients (% of the total)**	
1–2 cm	15 (3.4)
2–3 cm	114 (25.9)
3–5 cm	273 (62.2)
>5 cm	37 (8.5)
**Echogenicity, number of patients (% of the total)**	
Hypoechoic, regular	109 (25)
Hypoechoic, irregular	72 (16.4)
Mixed (hypo- and hyperechoic), regular	165 (37.6)
Mixed (hypo- and hyperechoic), irregular	93 (21)

No significant differences were found between the first operator and the second blinded examiner (*p* = 0.122).

Chest HRCT was performed in 239 patients (54.4% of the total), as needed for the final diagnosis. All the aforementioned findings assessed by LUS were confirmed by chest HRCT, when performed. Pleural effusion and pulmonary consolidation were detected in 56.2 and 98.7% of patients by HRCT, respectively (*p* = 0.211 and 0.101 vs. LUS, respectively).

There was no match between detection of the hyperehoic spots by LUS and the air bronchogram visualized by chest HRCT (*p* = 0.139).

**Figure 2:**
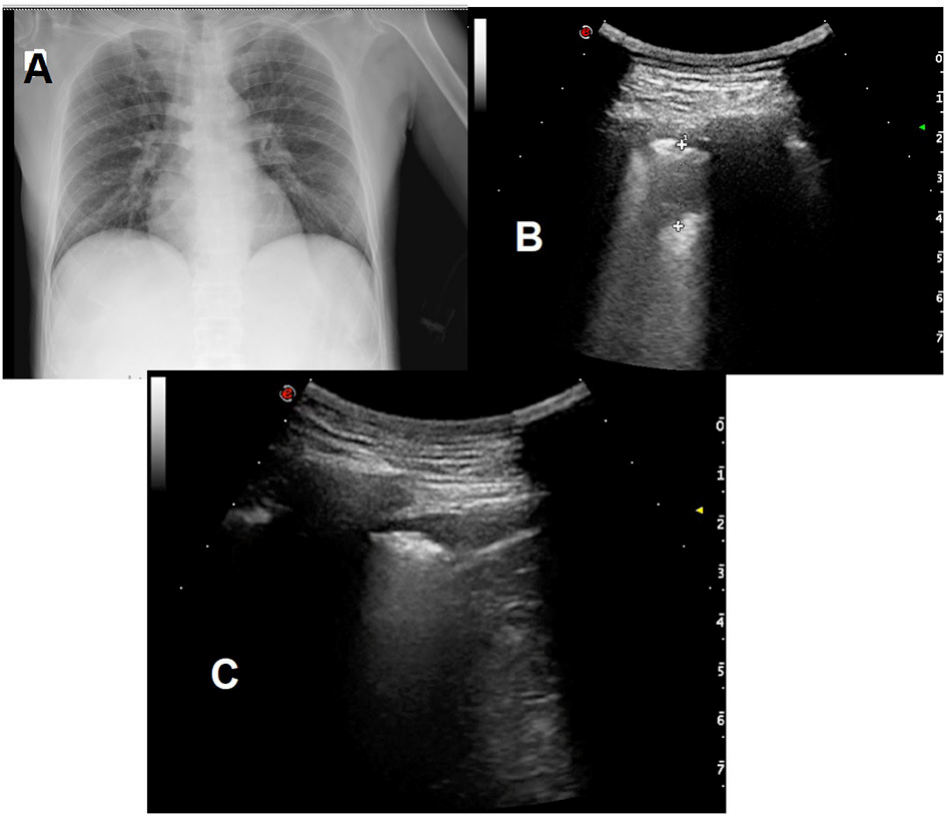
**(A)** Antero-posterior bedside X-ray showing no pulmonary consolidation and no apparent pleural effusion. **(B)** Posterior left basal LUS scan showing a small subpleural thickening of 1.5 cm. **(C)** hypoechoic consensual homolateral pleural effusion (depth: 9 mm) in the diaphragmatic costophrenic sinus.

Figure 2 illustrates antero-posterior bedside X-ray and LUS images of a patient. The chest X-ray was negative for pulmonary consolidation and pleural effusion, while LUS showed a small subpleural thickening and hypoechoic consensual pleural effusion.

## Discussion

The systematic use of bedside LUS, in association with clinical guidelines for the diagnosis of pneumonia, is clinically useful in the diagnostic evaluation of acute dyspnea. In particular, LUS is accurate in the detection of pleural effusion and lung consolidation in patients with community-acquired pneumonia.

Our findings are in line with the well-known performance of LUS in visualizing even small amount of liquid, not visualized by chest X-ray (15, 19). The results demonstrate that it is possible to make a real time differential diagnosis between pulmonary consolidation and pleural effusion by LUS in cases of opacity detected by chest X-ray. Additionally, both pleural effusion and lung consolidation may be visualized by LUS, thus increasing the accuracy of the diagnosis.

Consolidations due to infective lung disease are mostly visualized as mixed, hypo- and hyperechoic, or hypoechoic, images by LUS, with regular margins, and associated with pleural effusion in about half of the cases. Hyperechoic spots, defined by some authors as “air bronchogram,” may be detected within lung consolidation by LUS in a significant percentage of cases, as well (20). In this context, it is important to underline that this hyperechoic images does not match to the chest HRCT finding of the air bronchogram, as properly defined (21). Hence, operators should not be misled by the visualization of these hyperechoic spots within the lesions by LUS, as their clinical significance is not comparable to what observed by chest HRCT. This misleading may indeed lead to errors and confusion in the differential diagnosis (21).

As in many other pleuropulmonary disorders, B lines visualized by LUS are increased in patients with acute dyspnea from infective lung disease (9). These data exclude the specificity of B lines in the differential diagnosis of dyspnea. Previous data from our group demonstrated no substantial role of B lines assessment by LUS in the differential diagnosis of dyspnea across a wide spectrum of pleuropulmonary and cardiac diseases (9). Several factors may cause the increase in the number of B line artifacts, and are mostly associated with the difference in acoustic impedance existing in normal lungs that is increased by the pathophysiological mechanisms of the underlying pleuropulmonary disease (9, 22). Considering the high percentage of patients with comorbidities in our study, these mechanisms are further reinforced. In this context, we have recently observed that no B lines are detectable by LUS when the difference in acoustic impedance between the chest wall and the lungs is removed, as during video-assisted thoracic surgery (23).

The limitation of our study is the lack of comparison with the gold standard chest HRCT in all patients, particularly considering the limitation of ultrasound in the emergency setting (24). Notwithstanding, the diagnosis was made in all patients in accordance with IDSA/ATA consensus guidelines for the diagnosis of community-acquired pneumonia comprising the assessment of clinical, laboratory, and imaging findings (2007, revised in 2019) (18, 25). Additionally, our primary aim was to describe the clinical utility of bedside LUS in patients presenting with dyspnea from infective lung disease and provide clinicians with information on the LUS semeiotics and findings and how they should be interpreted in these cases.

In conclusion, LUS represents a useful complementary tool, in association with clinical, laboratory, and radiological workup, as defined by clinical guidelines, in patients with dyspnea from infective lung disease. The technique is particularly useful in the differential diagnosis of pleural effusion vs. lung consolidation in cases of opacities visualized by chest X-ray, or in cases of negative radiology when clinical suspicious is high. Consolidations from infective lung disease have mostly mixed echogenicity by LUS, and are associated with pleural effusion in about half of the cases.

## Data Availability Statement

The datasets generated/analyzed for this study are not publicly available but are available from the corresponding author on reasonable request.

## Ethics Statement

The protocol was approved by the Ethics Committees of all the centers participating in the study. The patients/participants provided their written informed consent to participate in the study.

## Author Contributions

PB contributed to design, data collection, drafting, and revision of the manuscript. SB contributed to data collection and revision of the manuscript. CC contributed to data analysis, drafting, and revision of the manuscript. All authors have revised and accepted the present version of the manuscript.

## Conflict of Interest

The authors declare that the research was conducted in the absence of any commercial or financial relationships that could be construed as a potential conflict of interest. The handling editor declared a past collaboration with one of the authors CC.

## Publisher's Note

All claims expressed in this article are solely those of the authors and do not necessarily represent those of their affiliated organizations, or those of the publisher, the editors and the reviewers. Any product that may be evaluated in this article, or claim that may be made by its manufacturer, is not guaranteed or endorsed by the publisher.
